# Assessing Müllerian mimicry in North American bumble bees using human perception

**DOI:** 10.1038/s41598-022-22402-x

**Published:** 2022-10-20

**Authors:** Joseph S. Wilson, Aaron D. Pan, Sussy I. Alvarez, Olivia Messinger Carril

**Affiliations:** 1 Department of Biology, Utah State University - Tooele, Tooele, UT 84074 USA; 2 Museum of Texas Tech University, Lubbock, TX 79409 USA; 3 Santa Fe, NM 87540 USA

**Keywords:** Evolutionary ecology, Batesian mimicry, Mullerian mimicry

## Abstract

Despite the broad recognition of mimicry among bumble bees, distinct North American mimicry rings have yet to be defined, due in part to the prevalence of intermediate and imperfect mimics in this region. Here we employ a generalization approach using human perception to categorize mimicry rings among North American bumble bees. We then map species distributions on North American ecoregions to visually test for geographic concordance among similarly-colored species. Our analyses suggest that there are five mimicry rings in the North American bumble bee mimicry complex, and one broadly distributed group of mixed and intermediate color forms. We describe the Black Mimicry Ring, Black-cloaked Mimicry Ring, Eastern Yellow Mimicry Ring, Red Mimicry Ring, and Western Yellow Mimicry Ring as well as the mixed group. We then test these hypothesized mimicry rings by examining other insects that participate in these mimicry rings. Describing these mimicry rings is a vital step that will enable future analyses of imperfect mimicry, intermediate mimicry, and additional analyses of other insects that mimic bumble bees.

## Introduction

Color mimicry is often celebrated as one of the most recognizable outcomes of natural selection. Perhaps the most familiar type of mimicry is the concept of Batesian mimicry, where a harmless organism has evolved to have a similar appearance to a harmful species to avoid predation^1^. Müllerian mimicry, on the other hand, where two or more sympatrically occurring harmful species share a common color pattern^2^, is less well-understood. Among the best documented Müllerian mimicry complexes are the neotropical *Heliconius* butterflies^3–6^, in which around 25 different mimetic races occur between two species. Velvet ants are also known to participate in large Müllerian mimicry complexes in North America^7,8^ and Africa^9^. Bumble bees in the genus *Bombus* also have been shown to share aposematic patterns and are known to form multiple mimicry complexes worldwide (e.g.,^10–14^).

While mimicry in bumble bees has been recognized for over a century (e.g.,^15–17^), recent analyses have shown how complicated this mimicry system is. For example, detailed morphological analyses of 260 bumble bee species across the world found at least 400 distinct color patterns^11^, with many of these patterns being geographically restricted, suggesting Müllerian mimicry is occurring. Furthermore, analyses of polymorphic species have also found evidence of cryptic speciation and Müllerian mimicry (e.g.,^12,13^). Recent machine learning-based analyses that quantified and modelled predator perception found four mimicry groups among North American bumble bee species^14^. This powerful approach, however, revealed that mimicry in bumble bees involves a great deal of morphological variation and geographical overlap among phenotypes, with broad transition zones between color pattern mimicry groups that implies imperfect mimicry in intermediate zones^14^.

Another factor complicating the analyses of mimicry in bumble bees is the high degree of polymorphism, color variation, and body size among individuals and populations. Often, researchers use standardized templates to visualize color patterns and test for similarities among species while controlling for body size and orientation^11–14,18,19^. These standardized templates, while useful in making comparisons among species, are limited in that they do not always represent what species look like in the field (Pers. Obs.). Therefore, investigating mimicry using both standardized templates and images of live specimens in the field may provide additional insights into mimetic groupings.

It is clear that North American bumble bees participate in mimicry complexes, yet the morphological and geographic overlap and complexities of the mimicry has precluded the discrete description of the mimicry rings among them. Here we use a generalization approach to categorize North American bumble bee species into hypothesized mimicry rings. Mounting evidence suggest predators often generalize when viewing aposematic color patterns based on few key visual elements^20–23^. Furthermore, several studies have shown that human generalization is similar to the generalizations of natural predators^24–27^. In this study, we group phenotypically similar bumble bee species into mimicry rings based on human perception of both templates and field-based images of these bees, employing techniques used in other analyses of mimicry^7,8,25,28^. Then, we map species distributions on North American ecoregions to visually test for geographic concordance among similarly colored species as a way to define North American bumble bee mimicry rings. We then test these proposed mimicry rings by examining other non-bumble bee participants in these mimicry rings, including both Müllerian participants (other bee species) and Batesian mimics (various fly mimics).

## Results

### Bumble bee mimicry rings

**Figure 1:**
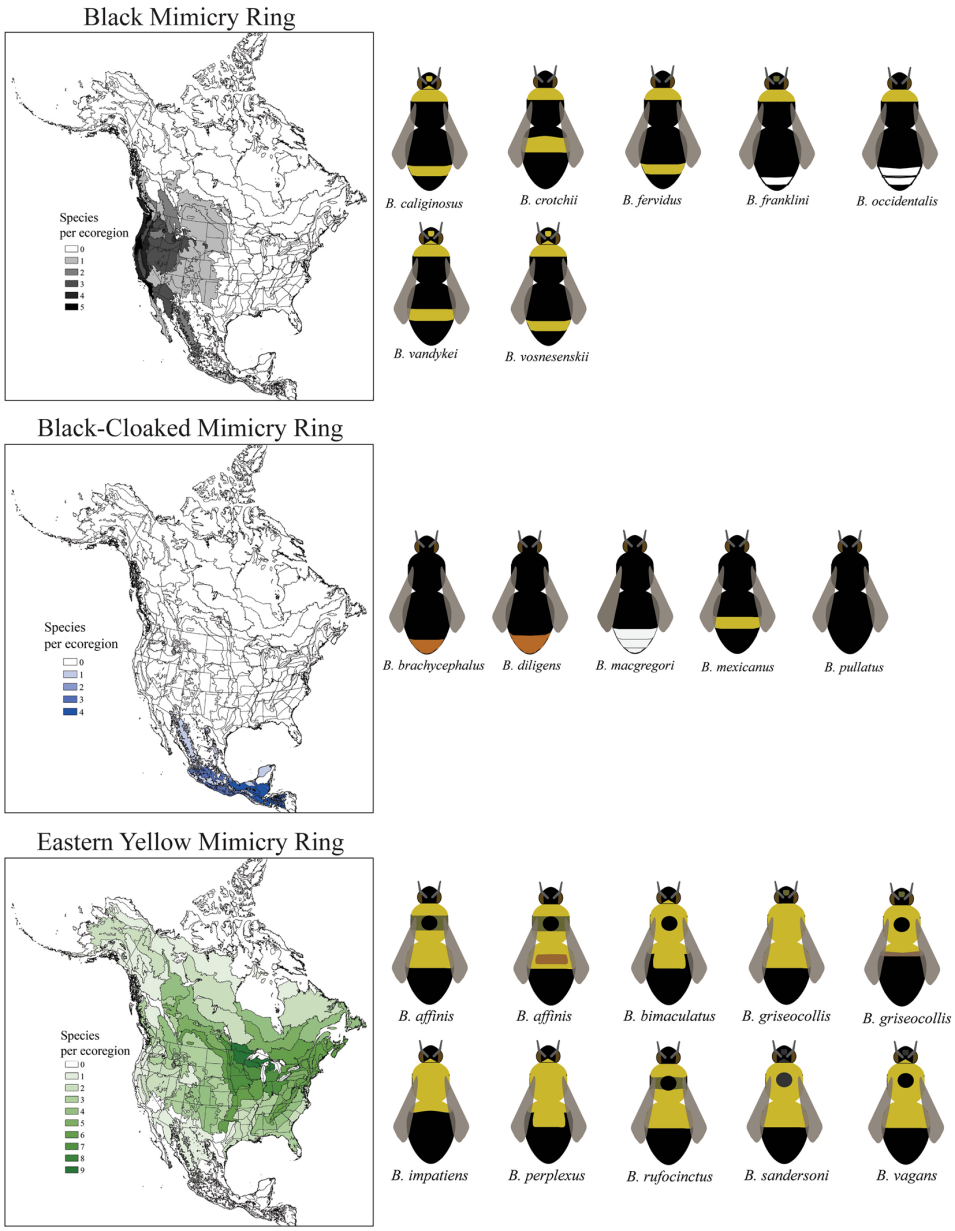
North American bumble bee mimicry rings. The morphological and geographic ranges of three of the five North American bumble bee mimicry rings. Here we present standardized color templates for each species involved in each of the mimicry rings. The geographic range of each mimicry ring is presented based on distributional analyses that examined the known range of each species projected onto North American ecoregions and mapped using ArcGIS version 10. This figure displays the Black Mimicry Ring, Black-Cloaked Mimicry Ring, and the Eastern Yellow Mimicry Ring.

**Figure 2:**
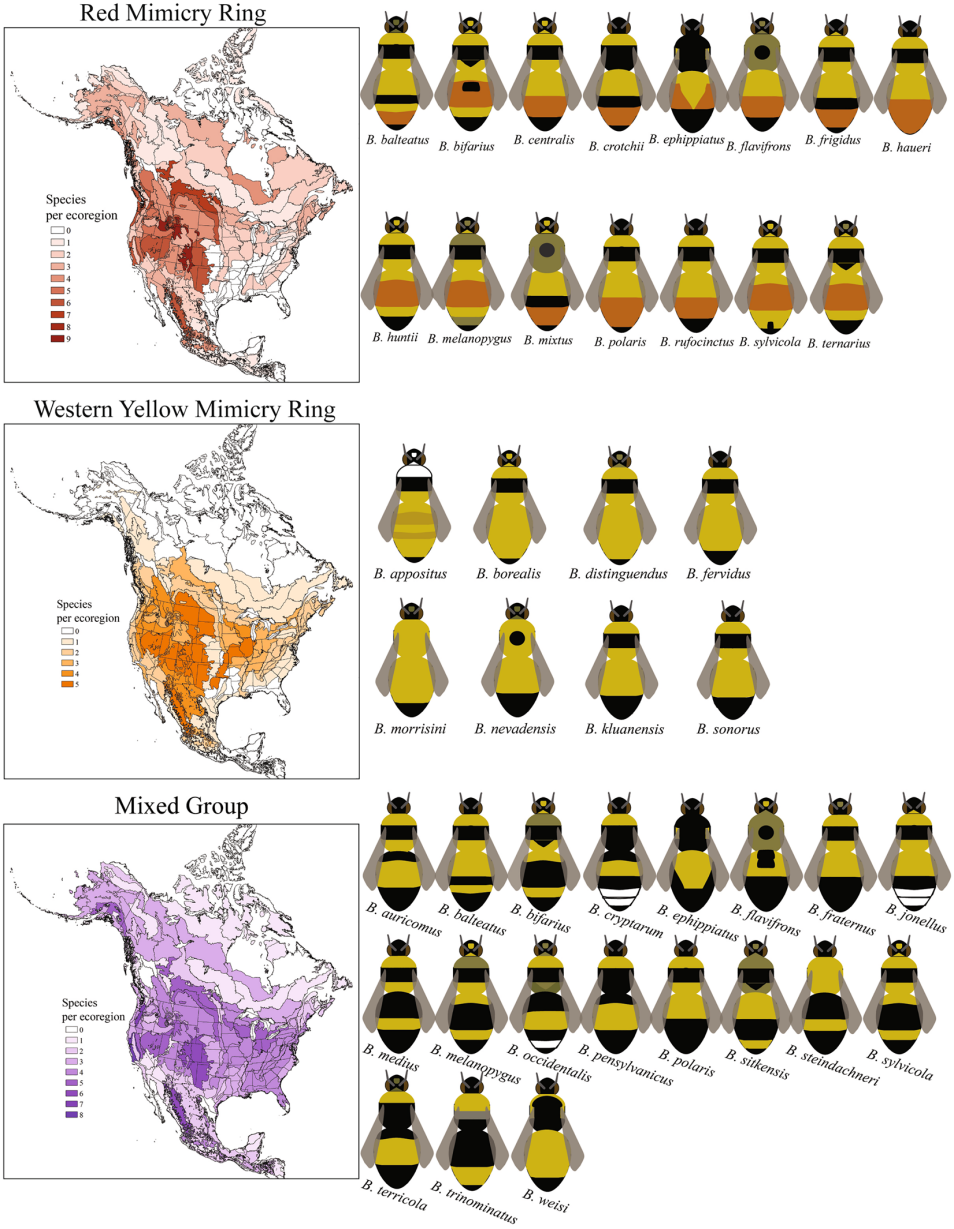
North American bumble bee mimicry rings. The morphological and geographic ranges of two of the five North American bumble bee mimicry rings as well as the mixed group. Here we present standardized color templates for each species involved in each of the mimicry rings and the mixed group. The geographic range of each mimicry ring is presented based on distributional analyses that examined the known range of each species projected onto North American ecoregions and mapped using ArcGIS version 10. This figure displays the Red Mimicry Ring, Western Yellow Mimicry Ring, and the Mixed Group.

Our analyses of visual similarity of female worker bumble bee color pattern templates and images indicate that there are five mimicry rings among the North American bumble bee mimicry complex, and one broadly distributed group of mixed and intermediate color forms (Figs. 1–2). We name these five mimicry rings based on the main color pattern shared between species and/or their common geographic distribution.

The Black Mimicry Ring contains seven species (Fig. 1) and is defined by the species having a large portion of their thorax and abdomen covered with black hair, particularly the scutellum and T1(the area connecting the thorax and abdomen) with yellow or white hairs forming secondary bands. The species in this ring, based on images of foraging workers, often look mainly black, with yellow secondary markings. *Bombus vosnesenskii* is a good example of a member of this mimicry ring. This mimicry ring is concentrated along the Pacific coast and Pacific northwestern parts of North America (Fig. 1).

The Black-cloaked Mimicry Ring contains five species (Fig. 1) and is defined by having the head, thorax and the majority of the abdomen covered in black hairs. The apical tip of the abdomen in many species is orange or pale yellow or even white. The species in this ring often appear to be all black because the vast majority of their bodies, especially the parts not obscured by the wings are entirely black. A good example of a member of this mimicry ring is *B. mexicanus*. This mimicry ring is found primarily in the tropical mountainous regions of southern Mexico, though a few species make it as far north as the Sierra Madre mountains of northern Mexico (Fig. 1).

The Eastern Yellow Mimicry Ring contains eight species (Fig. 1) and is defined by the species having a predominantly yellow thorax and the abdomen only being yellow near the base, with the apical abdominal sections being covered with black hair. While some species have some dark hairs on the thorax (and some occasionally have brownish hairs on the first abdominal section) the overall look of the species in this mimicry ring appear to be basically half black (mainly the abdomen) and half yellow (mainly the thorax and T1). *Bombus impatiens* is a good example of a member of this mimicry ring. This mimicry ring is widespread, though species with this color pattern are most diverse in the upper midwestern and eastern portions of North America (Fig. 1).

The Red Mimicry Ring contains 15 species, all of which have orange or red hairs on their abdomens, most of them forming a band (Fig. 2). The species in this ring are primarily yellow with red and black patterns on the abdomen, and most species also have black hair bands on the thorax. Members of this mimicry ring can be easily recognized by the presence of red/orange hair on their abdomens. *Bombus huntii* is a good example of a member of this mimicry ring. This mimicry ring, while widespread, is concentrated in the western montane and intermountain ecoregions of North America (Fig. 2).

The Western Yellow Mimicry Ring contains eight species and is defined by the thorax and abdomen being mainly covered with yellow hairs with some black hairs near the tip of the abdomen and often forming a band on the thorax (Fig. 2). These species generally look yellow as they forage. *Bombus fervidus* is a good example of a member of this mimicry ring. This mimicry ring is widespread, though species with this color pattern are most common in western and midwestern parts of North America (Fig. 2).

**Figure 3:**
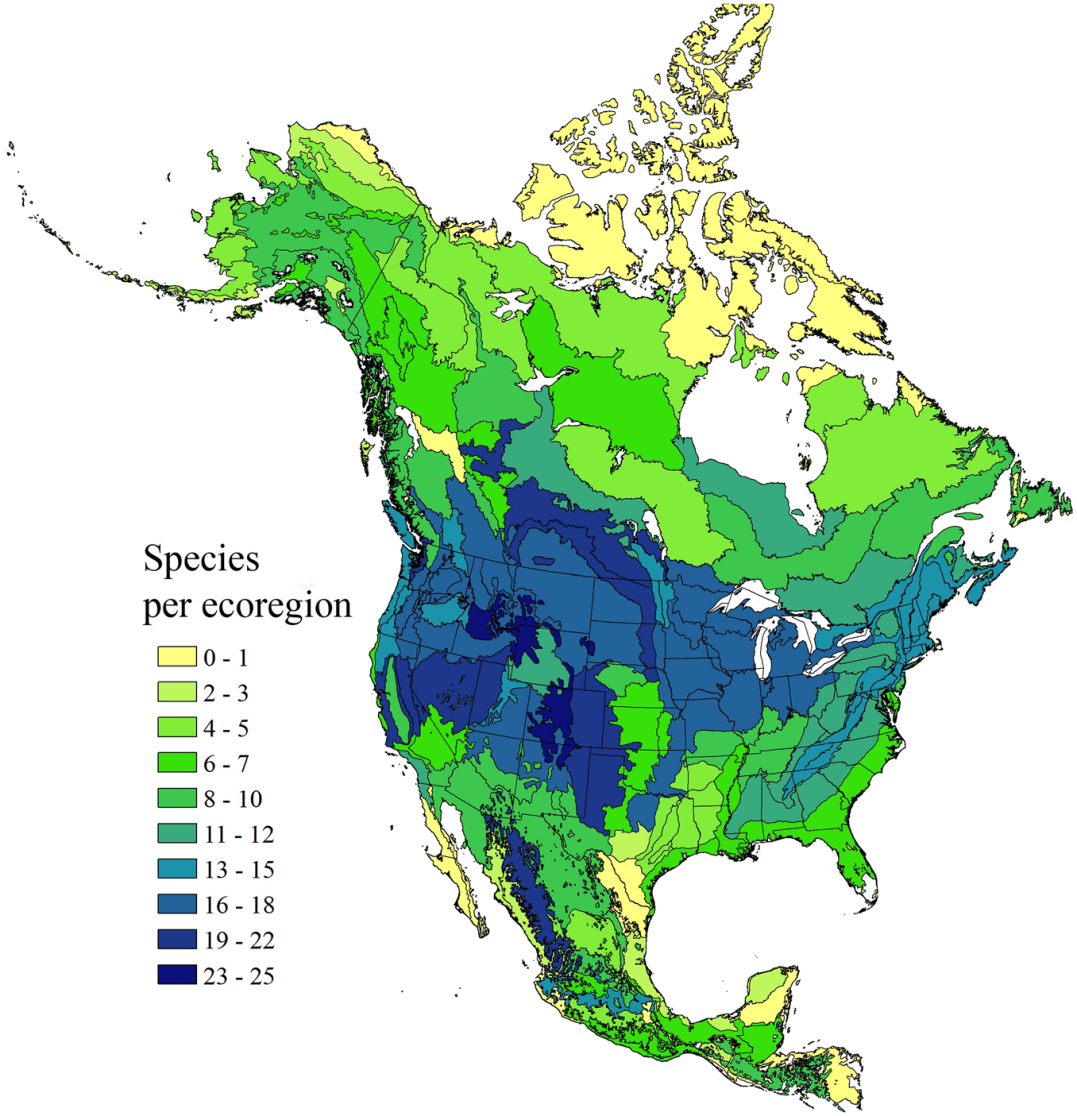
North American bumble bee richness. This map shows the species richness across North American ecoregions with darker colors indicating higher richness. This map was created using ArcGIS version 10.

The mixed group is not considered a distinct mimicry ring as there is a wide variety of color patterns and no geographic concordance (Fig. 2). This group contains 19 species and is phenotypically diverse and may contain many imperfect mimics or intermediate color patterns. Members of this group often have the thorax and abdomen a mixture of yellow bands and black bands. Species in this group generally have the look of a black and yellow striped bee. This group is widespread across North America with species diversity patterns mirroring overall species richness in North America (Figs. 2–3). Some of the species assigned to this group can look similar to the Eastern Yellow Mimicry Ring (e.g., *B. fraternus*) but the thorax of the members of the mixed group generally have a more pronounced black band. *Bombus pensylvanicus* is a good example of a member of this mimicry group.

### Non-bumble bee mimics

**Figure 4:**
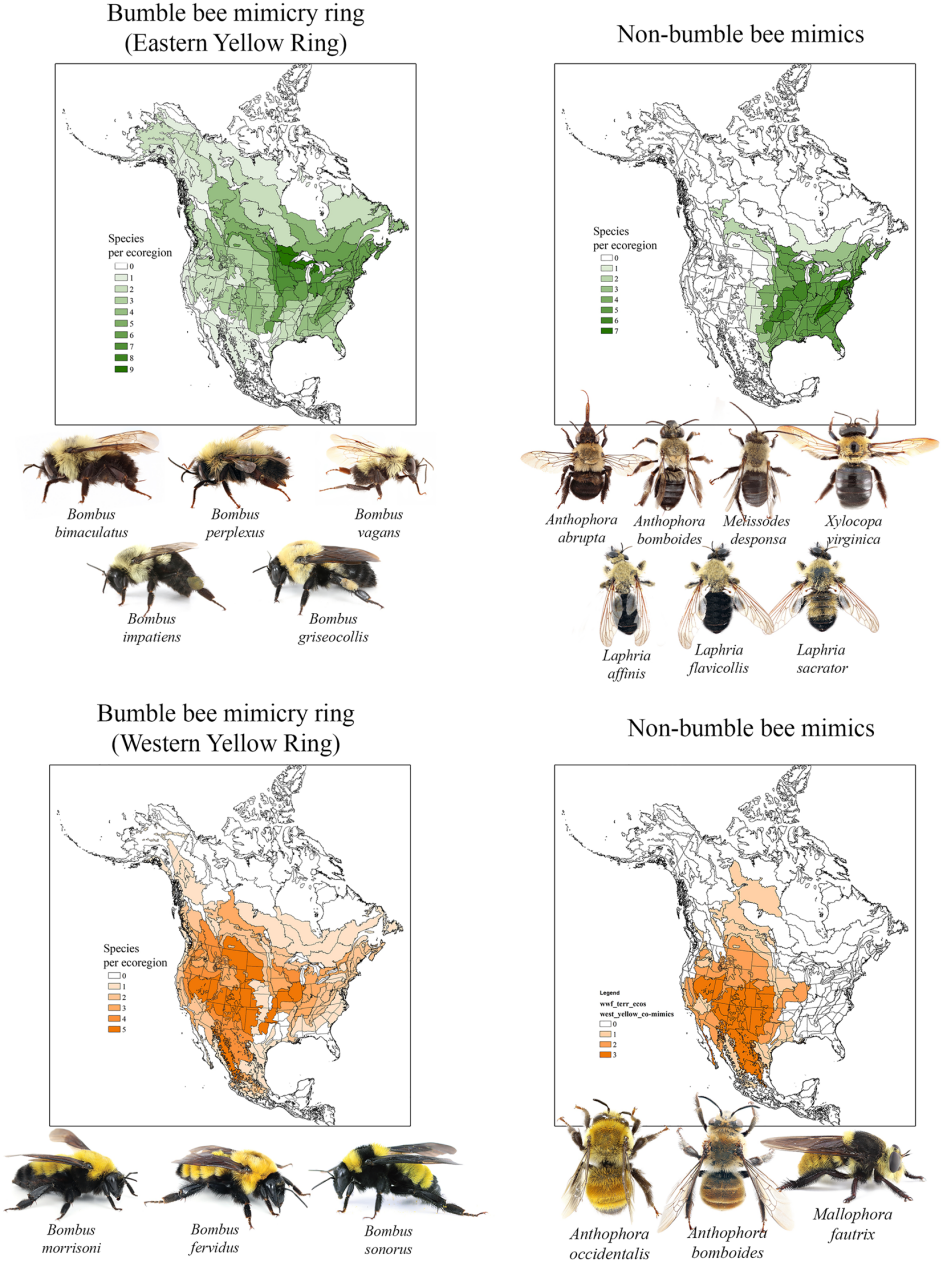
Bumble bee co-mimics. In addition to the Mullerian mimicry exhibited among bumble bee species, several other non-bumble bee insects participate in these large mimicry rings, some as Batesian mimics and others as Mullerian mimics. Two groups of non-bumble bee insects are presented in comparison to two of the bumble bee mimicry rings, the Eastern Yellow Ring and the Western Yellow ring. Several solitary bee species, as well as various robber fly species appear to participate in these mimicry rings bases on shared coloration and similar geographic ranges. See Fig S1 for more examples. Maps were created using ArcGIS version 10.

The phenotypic patterns observed in many non-bumble bee taxa lends support to our proposed mimicry rings as they appear to participate as co-Müllerian mimics. For example, in the widespread digger bee species *Anthophora bomboides*, populations living sympatrically with our proposed Eastern Yellow Mimicry Ring have a similar color pattern to this mimicry ring, i.e., they have a yellow head and thorax with a black abdomen (Fig. 4). However, *A. bomboides* populations living in the western U.S., sympatric with the Western Yellow and Red Mimicry Rings have a phenotype that is similar to these mimicry rings (Fig. 4). Lastly, some *A. bomboides* populations in the Pacific Northwest and Pacific coastal areas have a darker phenotype, similar to the Black Mimicry Ring (Fig. S1). As another example, the sister species *Anthophora abrupta* and *A. occidentalis* also phenotypically match the Bombus mimicry rings in each or their respective ranges, with *A. abrupta* having a similar color pattern to eastern bumble bees, and *A. occidentalis* resembling members of the Western Yellow Ring (Fig. 4). Many other widespread genera have eastern species that have the same color patterns as members of the Eastern Yellow Ring, while other members of the genus do not appear to mimic bumble bees. For example, multiple *Melissodes* species that live in the eastern parts of North America resemble members of the Eastern Yellow Ring, while most western species do not seem to mimic bumble bees (Fig. 4). Eastern carpenter bees (*Xylocopa virginica*) also appear to mimic eastern bumble bees, while their western relatives do not (Fig. 4). Finally, several flies that are thought to mimic bumble bees as Batesian mimics show similar geographic patterns with their color patterns to our proposed bumble bee mimicry rings. For example, several members of the robber fly genera *Laphria* and *Mallophora* have color patters that correspond to our proposed mimicry rings (Fig. 4). While there are numerous other groups of flies that also are considered bumble bee mimics, including many Syrphidae, and some Oestridae, we only investigated the color and distributional similarities of a limited number of robber flies as a way to test our proposed mimicry rings. Future studies should investigate other potential participants in the large mimicry complex including other flies, beetles, and moths. We are aware that we focused here on a handful of examples that do support our mimicry rings and that many other insects do not imitate the color patterns of bumble bees. However, it is fascinating and noteworthy that so many non-bumble bees do seem to support the proposed mimicry rings.

## Discussion

While mimicry among bumble bees is well established, this study is the first to treat and categorize all North American species (including those in Mexico) into five phenotypically distinct and geographically delimited mimicry rings and one mixed group (Figs. 1–2). Our groupings largely agree with those suggested by other researchers (e.g.,^10–14^). For example, many authors have pointed to the fact that several species living along the Pacific coast have a black color pattern and that species near the Rocky Mountains often have red (e.g.,^14,19,29^). However, our analyses do suggest that there are distinct eastern and western yellow mimicry rings and we also describe a new mimicry ring in Mexico.

Why is an analysis like this needed if mimicry is already well established among bumble bees? It is important to define mimicry rings among bumble bees for multiple reasons. First, bumble bees provide a good opportunity to study the evolution of imperfect mimicry. However, analyses of imperfect mimicry are not possible without first defining mimicry rings. Second, by describing generalized mimicry rings, future analyses can investigate how other taxa participate in these mimicry rings, both as Müllerian mimics and as Batesian mimics. Also, the description of these mimicry rings can allow for studies investigating the phylogenetic relatedness among mimicry rings to determine how much of the similarity is due to convergent evolution rather than common ancestry. Lastly, defining mimicry rings is useful in analyses of the evolution of polymorphism in bumble bee species.

We recognize that there is a great deal of geographic and phenotypic overlap between these mimicry rings, as has been shown in other analyses^14^. This overlap provides a unique opportunity to study imperfect and intermediate mimicry in geographic areas where multiple models co-occur. Furthermore, the large group of species we call the mixed group allows for investigations of selection on coloration. For example, 70% of the polymorphic species in our analyses had one morph belonging to the mixed group. Population genetic analyses and ancestral state reconstructions might be able to address whether the mixed group ring is an ancestral color pattern, or if it represents an intermediate (and selected for) color pattern.

We acknowledge that our methods for defining these mimicry rings likely oversimplifies some of the color variation seen among many bumble bee species. For example, we only included one color pattern of *B. nevadensis* in our analysis, which was placed in the Western Yellow Mimicry Ring. There are some known color forms of *B. nevadensis*, however, that might fit in the Red Mimicry Rings, or even the mixed group. Our analysis was designed to look for broad patterns among bumble bees in North America, not necessarily to look at all the exceptions to these broad patterns. We suggest that future analyses should look more closely at the specific population-level color patterns in widespread, polymorphic species, particularly in relation to the broad patterns we describe here. Other analyses should also investigate color patterns in male bumble bees or queens to compare those patterns to the mimicry rings we describe here. There is also a possibility that some of the color variation in bumble bees could be associated with environmental factors rather than only a product of mimetic evolution as has been seen in some velvet ant species^30^.

There are several aspects of bumble bee biology that could be related to the large amount of color variation both within mimicry rings, and even within species. Unlike other large mimicry complexes like those described in velvet ants^7–9^ and neotropical *Heliconius* butterflies^3–6^, bumble bees are unique in that they are social with caste differentiation within a colony (i.e., queen vs worker). This social structure likely results in different selective pressures on reproductive queens compared to non-reproductive workers. It is possible that selection on coloration is relaxed among the worker castes, which could be one reason for the large amount of imperfect mimicry in bumble bees. There is still much to be learned about mimicry in bumble bees, but we are hopeful that our definitions of broad mimicry rings will serve as an important springboard for future analyses that analyze both the exceptions to the mimicry patterns we have laid out, and the causes for the similarities between these bumble bee species.

## Conclusion

Our analyses suggest that North American bumble bees participate in a large mimicry complex with five distinct mimicry rings and one mixed group. These five rings, while not based on a precise model, are morphologically distinct and can be geographically defined. The mixed group provides further opportunities to investigate how mimicry, especially imperfect mimicry, evolved in bumble bees. The description of these five mimicry rings will allow for future analyses investigating non-bumble bee taxa that participate in this mimicry complex via Müllerian and Batesian mimicry.

## Materials and methods

### Identifying mimicry patterns

As has been done in previous studies, we used standardized templates (Figs. 1–2) colored to represent species-specific color patterns based on Williams et al.^19^ to investigate similarities among bumble bee species^11,14^. Color patterns of workers were used, as they are often the most common in an environment. For monomorphic species (e.g., *Bombus huntii*) only one template was used. For polymorphic species (e.g., *Bombus bifarius*) we used two templates representing the most divergent common color forms found in these species (Figs. 1–2). Because standardized templates were not available for Mexican species, we created our own based on the coloration of these species. We excluded cuckoo bumble bees in the subgenus *Psithyrus* from the analysis as they often do not have a consistent color pattern and do not represent a common component of a bumble bee community.

Prior knowledge of species and distributions can cloud people’s perceptions of visual similarity (i.e., if a researcher knows a species lives in a specific region, they might be more likely to subconsciously classify it as similarly colored to other species from that region). We avoided these effects by having freshmen biology students at Utah State University (N = 45) with little or no prior bumble bee identification experience group species templates based on similarities among color patterns. To do this, we first introduced the concept of mimicry to students and explained how predation drives the evolution of mimicry. Then we provided bumble bee species templates to students and instructed them to “think like a predator” and place bumble bee templates into similar looking groups. The idea was presented that a predator only has a very limited time to decide to attack or avoid a potential prey item so as they sort the bumble bee templates, they should imagine they are seeing the color patter only briefly and they need to decide if it looks like other color patters that they see on the table. While species groupings varied from student to student, this exercise enabled us to find broad patterns. For example, most students grouped species together that had a red/orange coloration on the abdomen. Similarly, most students grouped the species that are primarily covered in black hair together.

We then used these initial rough groups that were consistently made by students to further clarify and refine mimicry groups on our own by examining images of live species and distributional data. For example, students placed all yellow bumble bees together but after examining the geographic ranges of species, we further divided yellow bumble bees into the eastern yellow ring and the western yellow ring. Live images and distributional data were obtained through Discoverlife.org^31^ and iNaturalist.org^32^. Images from iNaturalist were filtered to only include “research grade” specimens (i.e., specimens that were identified by two or more experts), which included hundreds of images of bumble bees. In some instances, the live specimens did not look as similar to a specific group as the standardized template did. In these cases, we placed species into the mimicry group that the live specimen photos seemed to be more similar to. For example, the *B. affinis* template show the central brownish band on T1 (the first metasomal segment). In the template, this species looks similar to others that have red patterns on the abdominal segments. In the majority of the images of specimens, however, *B. affinis* resembled other eastern species more often than it resembled species with red on their abdomen. There were several species that students failed to group consistently, and there was little or no geographic concordance among species distributions. This group of species we placed together in a group that we are not specifically considering a mimicry ring, rather a mixed group of loosely affiliated color forms that do not fit well into any of the defined mimicry rings.

### Mapping species distributions

To visualize geographic patterns of similarly-colored bumble bees, we mapped the distribution of each species onto the ecoregions of North America. Maps were made on ecoregions as these areas represent ecologically similar habitat and can help mitigate the problems of unequal collection effort across North America. To map monomorphic species (species with only one color pattern), we downloaded species specific collection locality data from GBIF^33^ and mapped these data points on an ecoregion map using ArcGIS version 10. If a species was found in an ecoregion, that ecoregion was shaded in, darker shades, therefore, indicate ecoregions that house multiple species. Polymorphic species could not be mapped in the same way as online collection records generally do not include a photograph of the specimen or a description of the color pattern. For polymorphic species we used iNaturalist.org to identify color patterns and collection localities for individuals. First, we filtered uploaded observations by species. Next we included only “research grade” species identifications (those that have been identified by 2 or more experts) in our search. We then examined the images associated with each observation record to determine which of our putative mimicry rings the individual belonged to. Observation locality data were then used to construct ecoregion maps for each broad morph of the polymorphic bumble bee species.

### Non-bumble bee mimetic species

To test our proposed mimicry ring hypotheses, we investigated a variety of other insect taxa that are often considered bumble bee mimics to see if the mimetic phenotypes and distributions corresponded to our proposed mimicry rings. Many other bee species have similar color patters to bumble bees. Because these other bees are also able to sting, we considered them Müllerian participants in the bumble bee mimicry rings. Non-bumble bees used to in the analysis include some widespread polymorphic species (*Anthophora bomboides*) and groups consisting of closely related species exhibiting different color patterns (*Anthophora abrupta* and *A. occidentalis*, *Xylocopa virginica* and other *Xylocopa* species, and various *Melissodes* species). We also investigated various fly species that are often considered Batesian mimics of bumble bees. These include some robber flies in the genera *Laphria* and *Mallophora*.

## Supplementary Information

Supplementary Information.

## Data Availability

The distributional data analyzed during the current study are publicly available on iNaturalist.org and Discoverlife.org. The phenotypic templates are available from Bumble Bees or North America by Williams, et al. 2014. Please send any data inquiries to the corresponding author.
