# Supplementary material for: Assessing Müllerian mimicry in North American bumble bees using human perception
Source: Sci Rep. 2022 Oct 20;12:17604. doi: 10.1038/s41598-022-22402-x (PMC9585094; doi:10.1038/s41598-022-22402-x)

**Assessing Mullerian mimicry in North American bumble bees using human perception**

*Joseph S. Wilson, Aaron D. Pan, Sussy I. Jones*, *and Olivia Messinger Carril*

**Figure S1. Additional Bumble bee co-mimics.**  In addition to the Mullerian mimicry exhibited among bumble bee species, several other non-bumble bee insects participate in these large mimicry rings, some as Batesian mimics and others as Mullerian mimics. Two groups of non-bumble bee insects are presented in comparison to two of the bumble bee mimicry rings, the Red Ring and the Black ring. Several solitary bee species, as well as various robber fly species appear to participate in these mimicry rings bases on shared coloration and similar geographic ranges. See Fig 4 for more examples.


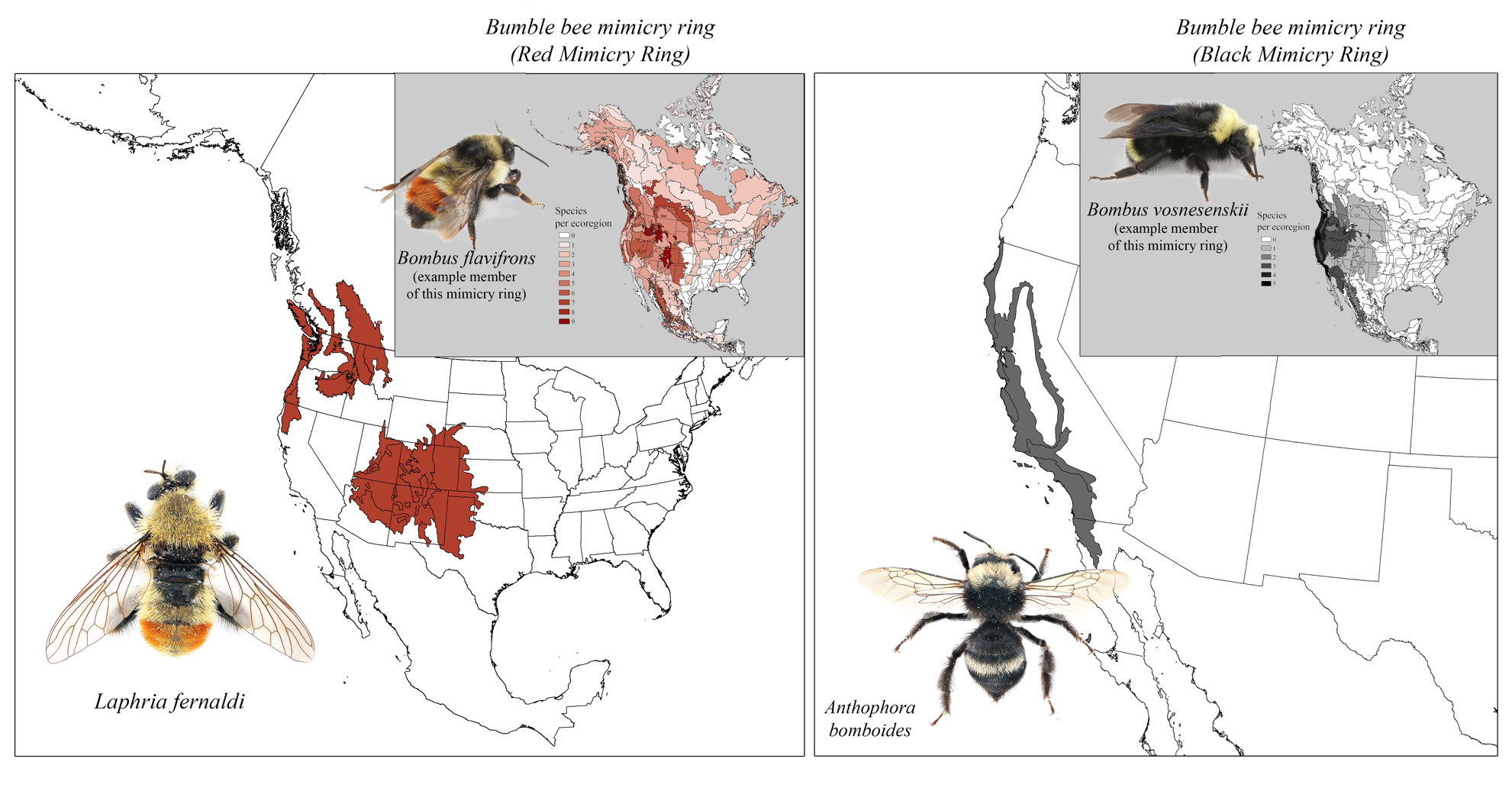

Supplement: Supplementary file 1 — Supplementary Information. [file 41598_2022_22402_MOESM1_ESM.docx]
